# Metabolomic Analysis of Platelets of Patients With Aspirin Non-Response

**DOI:** 10.3389/fphar.2019.01107

**Published:** 2019-10-10

**Authors:** Jiun-Yang Chiang, Sheng-Han Lee, Yen-Ching Chen, Cho-Kai Wu, Jing-Yuan Chuang, Shyh-Chyi Lo, Huei-Ming Yeh, Shih-Fan Sherri Yeh, Cheng-An Hsu, Bin-Bin Lin, Pi-Chu Chang, Chih-Hsin Chang, Hao-Jan Liang, Fu-Tien Chiang, Ching-Yu Lin, Jyh-Ming Jimmy Juang

**Affiliations:** ^1^Cardiovascular Center and Division of Cardiology, Department of Internal Medicine, National Taiwan University Hospital and National Taiwan University College of Medicine, Taipei, Taiwan; ^2^Graduate Institute of Clinical Medicine, National Taiwan University College of Medicine, Taipei, Taiwan; ^3^Institute of Environmental Health, College of Public Health, National Taiwan University, Taipei, Taiwan; ^4^College of Public Health, Institute of Epidemiology and Preventive Medicine, National Taiwan University, Taipei, Taiwan; ^5^Department of Medical Laboratory Science and Biotechnology, China Medical University, Taichung, Taiwan; ^6^Department of Laboratory Medicine, National Taiwan University Hospital, Taipei, Taiwan; ^7^Department of Anesthesiology, National Taiwan University Hospital and National Taiwan University College of Medicine, Taipei, Taiwan; ^8^Department of Environmental and Occupational Medicine, National Taiwan University Hospital and National Taiwan University College of Medicine, Taipei, Taiwan; ^9^Division of Haematology, Department of Laboratory Medicine, National Taiwan University Hospital, Taipei, Taiwan; ^10^Cardiovascular Center, Fu-Jen Catholic University Hospital, New Taipei, Taiwan

**Keywords:** aspirin non-response, metabolomics, PFA-100, glycine, platelet

## Abstract

**Background:** Aspirin is the most commonly used antiplatelet agent for the prevention of cardiovascular diseases. However, a certain proportion of patients do not respond to aspirin therapy. The mechanisms of aspirin non-response remain unknown. The unique metabolomes in platelets of patients with coronary artery disease (CAD) with aspirin non-response may be one of the causes of aspirin resistance.

**Materials and Methods:** We enrolled 29 patients with CAD who were aspirin non-responders, defined as a study subject who were taking aspirin with a platelet aggregation time less than 193 s by PFA-100, and 31 age- and sex-matched patients with CAD who were responders. All subjects had been taking 100 mg of aspirin per day for more than 1 month. Hydrophilic metabolites from the platelet samples were extracted and analyzed by nuclear magnetic resonance (NMR). Both 1D ^1^H and 2D J-resolved NMR spectra were obtained followed by spectral processing and multivariate statistical analysis, such as partial least squares discriminant analysis (PLS-DA).

**Results:** Eleven metabolites were identified. The PLS-DA model could not distinguish aspirin non-responders from responders. Those with low serum glycine level had significantly shorter platelet aggregation time (mean, 175.0 s) compared with those with high serum glycine level (259.5 s). However, this association became non-significant after correction for multiple tests.

**Conclusions:** The hydrophilic metabolic profile of platelets was not different between aspirin non-responders and responders. An association between lower glycine levels and higher platelet activity in patients younger than 65 years suggests an important role of glycine in the pathophysiology of aspirin non-response.

## Introduction

Cardiovascular diseases are the most common cause of mortality and morbidity worldwide. According to the American Heart Association’s updated report in 2018, cardiovascular mortality ranked first in all kinds of deaths and produce immense health and economic burdens (Wu et al., 2010;Benjamin et al., 2018). In Taiwan, cardiovascular disease ranked second in the top 10 leading causes of death in 2011. Atherothrombosis, characterized by atherosclerotic lesion disruption with thrombotic complications, is the main cause of acute cardiovascular events. Accumulation of platelets at the site of atherosclerotic lesion disruption is the first step in the formation of atherothrombosis; these elements are responsible for the formation of pathogenic thrombi in patients with atherothrombotic diseases, such as coronary artery disease (CAD), ischemic stroke/transient ischemic attack (TIA), and peripheral artery disease (PAD).

Aspirin is by far the most commonly used antiplatelet agent for the prevention of cardiovascular diseases. The Antithrombotic Trialists’ Collaboration documented a 22% reduction in death and serious ischemic vascular events with the use of antiplatelet therapy when compared with a placebo in their most recent meta-analysis of 287 randomized trials, comprising more than 200,000 patients (Antithrombotic Trialists' Collaboration, 2002). Although aspirin therapy is effective, 8% to 45% of patients do not respond to aspirin therapy as determined by different laboratory tests; these aspirin non-responders are at increased risk of thrombotic events (Eikelboom et al., 2002; Gum et al., 2003; Chen et al., 2004; Krasopoulos et al., 2008; Foussas et al., 2009; Frelinger et al., 2009; Li et al., 2014). Stejskal et al. reported that patients with acute coronary syndrome and aspirin non-response had an 88% incidence of recurrent cardiovascular events compared with 47% in aspirin responders (Stejskal et al., 2006). Gum et al. showed that aspirin non-response was significantly associated with an increased risk of major cardiovascular adverse event (24% vs 10%, hazard ratio 3.12) (Gum et al., 2003).

Platelets are circulating cell fragments that play a pivotal role in thrombosis. Although the platelet contains no nucleus, selective ligands for platelet membrane receptors activate their nuclear receptors and regulate platelet aggregation and activation. A number of nuclear receptors have been found recently to be present in human platelets, including receptors for sex steroids, and glucocorticoids, along with peroxisome proliferator-activated receptors (PPARs) and retinoid X receptors (RXRs) (Bishop-Bailey, 2009), implying that biological reaction and metabolic activity in human platelet are active and complex.

There are a number of available tests for assessing platelet function. PFA-100 is one of the most commonly used modalities worldwide. Shortened PFA-100 closure time under aspirin treatment was associated with more ischemic events in patients with myocardial infarction (Reny et al., 2008). One recent reviewed article showed a trend toward more death and major adverse cardiovascular events in PFA-100-defined aspirin non-responders. The cutoffs of PFA-100 range from 150 to 193 s in different studies (Dretzke et al., 2015). However, research on normal reference of PFA-100 from Asian population is relatively limited.

Metabolomic studies provide a rapid and high throughput screening tool for understanding the relationships between metabolic profiles and biological effects in responding to stimulation of genetic modulation, drugs, toxicants, and various kinds of disease. The low molecular weight metabolites such as sugar, amino acids, lipids, intermediates in metabolism, and degradation products of exogenous compounds are analyzed in the field of metabolomics. Like genomics and proteomics, metabolomics can be examined in different levels of biological systems such as cells (Ellis et al., 2011;Wu et al., 2017), tissues (Viant et al., 2006; Lindon et al., 2007), biofluids (Tiziani et al., 2009), and whole organisms. Nuclear magnetic resonance (NMR) is a common analytical technique applied in metabolomics; it requires less sample preparation and provides rapid analysis and high reproducibility of metabolic profiles. Similar to other omics, metabolomics can also be applied in several research fields such as pharmacology (Bollard et al., 2005), toxicology (Azmi et al., 2005; Viant et al., 2006), and disease diagnosis (Brindle et al., 2002; Gowda et al., 2008).

Several single nucleotide polymorphisms, miRNAs, increased inflammatory activity, drug noncompliance, and metabolic syndromes have been proposed as contributors to residual platelet activity under aspirin treatment (Wurtz et al., 2012; Weng et al., 2013; Du et al., 2016). For example, genetic polymorphisms in platelet glycoprotein and P2Y receptors are linked to an attenuated effect of aspirin, and increased risk of thromboembolic events (Szczeklik et al., 2000; Li et al., 2007). Most studies of platelet of aspirin non-responder focused on upstream biological processes, but few have addressed the downstream omics such as metabolomics. Research on platelet’s metabolites has revealed that certain metabolites were associated with platelet’s recovery after storage and its survival (Zimring et al., 2016), proving the concept that metabolomics may be a reflection of certain phenotype of platelet. By utilizing metabolomic analysis, changes in the small molecules of platelets in aspirin non-responders may be detected, providing insights into the mechanism of non-response, and potentially discovering new biomarkers for this population (Johnson et al., 2016).

We prospectively enrolled patients with CAD confirmed by coronary angiography or multidetector computed tomography (MDCT) who took aspirin daily and we then compared platelet metabolome between patients with CAD with and without aspirin non-response.

## Methods

### Subjects

This cross‐sectional case-control study included a total of 60 Taiwanese cardiovascular patients aged 39 to 85 years who had elective coronary angiography for CAD or valvular heart disease at National Taiwan University Hospital between June 2011 and February 2013. All enrolled subjects had been taking 100 mg of aspirin per day for more than 1 month. The study complies with the declaration of Helsinki, and the study protocol was approved by the institutional research ethics committee (IRB number: 201106077RC). All patients gave written informed consent. Each patient filled out a self‐report questionnaire and supplied blood samples for identification of aspirin users and further metabolomics research. A self‐report questionnaire was conducted to collect information on demographics, platelets count, disease history (i.e., hypertension, diabetes and fatty liver), lifestyle (i.e., smoking), and general biological parameters (i.e., hemoglobin, aspartate aminotransferase, creatinine, total cholesterol, triglycerides, sodium and potassium). Aspirin non-responder was defined as a study subject whose platelet aggregation time result was less than 193 s by PFA-100 test. Details of PFA-100 test are provided in the following section. For each aspirin non-responder, a responder, with PFA-100 result more than 193 s, was chosen matching on age, sex, diabetes mellitus, hypertension, dyslipidemia, smoking status, level of hemoglobin, platelet, renal function, liver function, and lipid profile as these factors are potential confounders of platelet function.

### Sample Collection and Preparation of Platelets

Blood samples were collected *via* radial or femoral artery using 18-gauge needles on the enroll day at the catheterization laboratory, and processed within 1 h after collection. Fresh blood (10 ml) anticoagulated with ethylenediaminetetraacetic acid (EDTA) was collected for baseline hemoglobin and platelet count, and 20 ml whole blood was collected in evacuated tubes containing 3.2% sodium citrate. The whole blood mixed with sodium citrate was centrifuged at 200 g for 20 min to obtain platelet-rich plasma (PRP). The platelets were prepared from PRP by centrifugation (900*g*, 10 min) and washed with Tyrode’s buffer ((137 mM NaCl, 2.65 mM KCl, 12 mM NaHCO_3_, 0.43 mM NaH_2_PO_4_, 2 mM CaCl_2_, 1 mM MgCl_2_, 5 mM glucose, 5 mM HEPES, pH 7.35) containing 0.5 μM PGI*_2_* and 0.2 unit/ml apyrase. Washed platelets were then re-suspended in Tyrode’s buffer and adjusted to 3 × 10^8^/ml for platelet aggregation (Chang et al., 2015).

### Assessment of Response to Aspirin Intake

Blood collected in 3.2% sodium citrate was tested by PFA-100 (Siemen Healthcare Diagnostics, Marburg, Germany). PFA-100 tests were performed within 1 h of blood sampling. Whole blood was loaded into prefabricated proprietary cartridges containing a combination of collagen and epinephrine as platelet agonists. Platelet function analyzer closure time (the time for cessation of flow caused by formation of a platelet plug) was recorded in seconds. The maximum time allowed for closure was 300 s. Because no normal ranges had been established in Asian populations, we conducted a study on 100 healthy voluntary controls (50 men and 50 women; mean age = 42.1 ± 10 years) who were not taking any medications; these individuals served as internal controls to define the normal range of platelet aggregation for Taiwanese people. The results showed 86 to 193 s for closure of Col/EPI cartridge, 61 to 109 s for Col/ADP cartridge. This cutoff value was similar to prior studies in Caucasian population (Gum et al., 2001; Jilma and Fuchs, 2001; Angiolillo et al., 2006; Simpson et al., 2014). The distribution of clotting times of aspirin responders and non-responders in our study was shown in **Supplementary Figure 1**. Participants with a clotting time greater than or equal to 300 s, the upper detection limit of PFA-100 assay, were grouped together.

### Sample Preparation for Metabolomic Study

Extraction of low molecular weight metabolites from the platelets was conducted as described in previous research (Lin et al., 2007). Briefly, metabolites were extracted by resuspending the platelets collected from fresh blood anticoagulated with EDTA (average 280*10^6^ platelet/ml) and precipitating platelet proteins in a total of 1.16 ml methanol-chloroform-water (2/2/1.8) solvent (Bligh and Dyer, 1959). After vortexing and centrifugation (4°C, 1,000*g*, 15 min), 0.6 ml hydrophilic metabolic extracts were taken and dried out with a speed vacuum and then stored at −80°C until NMR analysis.

### NMR Spectroscopy

Hydrophilic platelet metabolites were resuspended in a 600-µl D_2_O sodium phosphate buffer (0.1 M, pH 7.4) containing 0.25 mM 3-trimethylsilyl-2,2,3,3-d4-propionate (TSP) as an internal standard. NMR spectra were acquired from 500.13 MHz in Bruker Avance-500 spectrometers equipped with a 5-mm QNP CryoProbe (Bruker, BioSpin) at the Core Facility for Protein Structural Analysis supported by the National Core Facility Program for Biotechnology in Academia Sinica, Taiwan.

Both ^1^H and JRES NMR spectra were acquired at 300 K. ^1^H NMR spectra were acquired from the pulse sequence (relaxation delay: -90°-t-90°-tm-90°-acquired free induction decay) with presaturation of water resonance to suppress the water signal. For each sample, 32 k data points were collected from 128 scans in a 20-ppm spectral width with a 2.0-s relaxation delay and an acquisition time of 1.63 s. JRES NMR spectra were acquired from the pulse sequence (relaxation delay-90°-t1-180°-t1-acquired free induction delay, with water suppression during relaxation delay). Sixteen k data points in F2 frequency axis and 40 data points in F1 frequency axis were collected in eight scans with a 2.0-s relaxation delay. The spectral widths of the F2 and the F1 axis were set 6000 and 65 Hz, respectively.

### NMR Spectral Processing

^1^H NMR spectral data were zero-filled to 64 k points and processed with 0.3 Hz exponential line-broadening before Fourier transformation. The spectra were phased, and the baseline was corrected and calibrated by TSP (δ = 0.00 ppm) by Topspin software (Version 2.1; Bruker, Billerica, MA, USA). JRES spectra were zero-filled to 8 k data points in F2, and up to 128 increments in F1 and processed with 0.3 Hz exponential line-broadening in both dimensions prior to Fourier transformation. The spectra were tilted (45°), symmetrized by F1, skyline-projected first and then manually phased, and the baseline was corrected and calibrated as ^1^H NMR analysis.

The ^1^H and the J-resolved spectroscopy projections (p-JRES) data were further processed by custom-written ProMetab software (version ProMetab_v3_3) (Viant, 2003) located in the MATLAB platform (Version 7.8; The MathWorks, USA). Spectra were divided into 1,850 chemical shift bins by using a bin width of 0.005 ppm (2.5 Hz) between 0.2 and 10.0 ppm, and then integrated spectral intensity for each bin. The regions of internal standard and water resonance were excluded before being normalized by total spectral area. The binned data were adjusted by generalized log transformation and Pareto scaling prior to multivariate analysis (**Supplementary Table 1**) (van den Berg et al., 2006).

### Statistical Analyses and Metabolite Identification

The processed p-JRES data were uploaded to Metaboanalyst 2.0 (http://www.metaboanalyst.ca) (Xia et al., 2012) for principal component analysis (PCA) and partial least‐squares discriminant analysis (PLS‐DA) (Hastie et al., 2009; Dormann J Elith et al., 2013). These two analyses evaluated the similarities among samples and identified unique metabolites corresponding to specific clusters. PCA, an unsupervised method, was used to reduce the complexity of metabolomics data matrix without additional group information. The PCA score plot provided the visual performance of the metabolome variation for each sample. In contrast, PLS‐DA, a supervised method, linked the group information and NMR dataset to determine the variance between the aspirin-responders and aspirin non-responders. The PLS‐DA score plot was used to illustrate if the metabolome of different aspirin-response types could be separated. The loading plots of PCA and PLS-DA were employed to identify the possible metabolites corresponding with different aspirin groups. Cross‐validated predictive capability (Q^2^) in 10‐fold internal cross‐validation and statistic value *p* in permutation test were conducted to evaluate the robustness and predictability of the PLS‐DA models (Westerhuis et al., 2008). Wilcoxon signed-rank tests (continuous variables) and Pearson’s χ^2^ tests (categorical variables) were utilized to compare the basic demographic data between aspirin responders and non-responders. Since age status was regarded as a major factor for clinical study, a stratified analysis was conducted to assess the association between metabolites and aspirin according to the referring value (clotting time = 193 s) or the median of the clotting time. The possible relationship between the significant changed metabolite and clotting time was assessed by the spearman’s rank correlation test. All analyses were administered by using SAS 9.2 (SAS Institute, Cary, NC, USA). All statistical tests were two‐sided, and the value of significance was set at *p* < 0.05.

The platelet metabolites from the ^1^H NMR spectra were identified by using Chenomx NMR software suite (Professional Edition, Version 5.1; Chenomx, Inc.).

## Results

### Characteristics of the Study Population

A total 31 aspirin responders and 29 non-responders were enrolled in the study. There is no significant difference in mean age, distribution of sex, body mass index, serum level of hemoglobin, platelet, total cholesterol, triglyceride, and percentage of patients with hypertension and diabetes mellitus (DM) between aspirin responders and non-responders (**Table 1**).

**Table 1:** Characteristics of the study population.

Variables	Aspirin responder (*n* = 31)	Aspirin non-responder (*n* = 29)	*P*
Age (years)	63.5 ± 8.08	62.3 ± 12.83	0.66
Male (%)	23 (74.2%)	24 (82.8%)	0.42
Body weight (kg)	67.8 ± 13.48	74.6 ± 16.64	0.12
Body mass index (kg/m^2^)	26.0 ± 4.17	27.0 ± 4.80	0.34
Hemoglobin (g/dl)	13.5 ± 1.99	14.5 ± 1.90	0.06
Platelet (k/dl)	234.7 ± 45.54	232.2 ± 73.77	0.11
Aspartate aminotransferase (μl)	23.3 ± 9.23	25.7 ± 13.66	0.93
Creatinine (mg/dl)	1.2 ± 1.09	1.1 ± 0.38	0.58
Total cholesterol (mg/dl)	180.3 ± 30.35	174.5 ± 37.27	0.31
Triglycerides (mg/dl)	135.3 ± 79.60	138.3 ± 71.28	1.00
Sodium (mmol/µl)	139.6 ± 2.64	139.7 ± 2.35	0.75
Potassium (mmol/µl)	4.3 ± 0.41	4.3 ± 0.36	0.49
	n (%)		
Hypertension	20 (64.5)	19 (65.5)	0.94
Diabetes mellitus	18 (58.1)	12 (41.4)	0.20
Lipid	16 (51.6)	16 (55.2)	0.78
Ever smoker	5 (16.7)	8 (27.6)	0.37

Continuous variable is shown as mean ± SD.

OR, odds ratio; SD, standard deviation.

### Metabolic Variation of Patients by Multivariate Statistical Analysis

**Figure 1:**
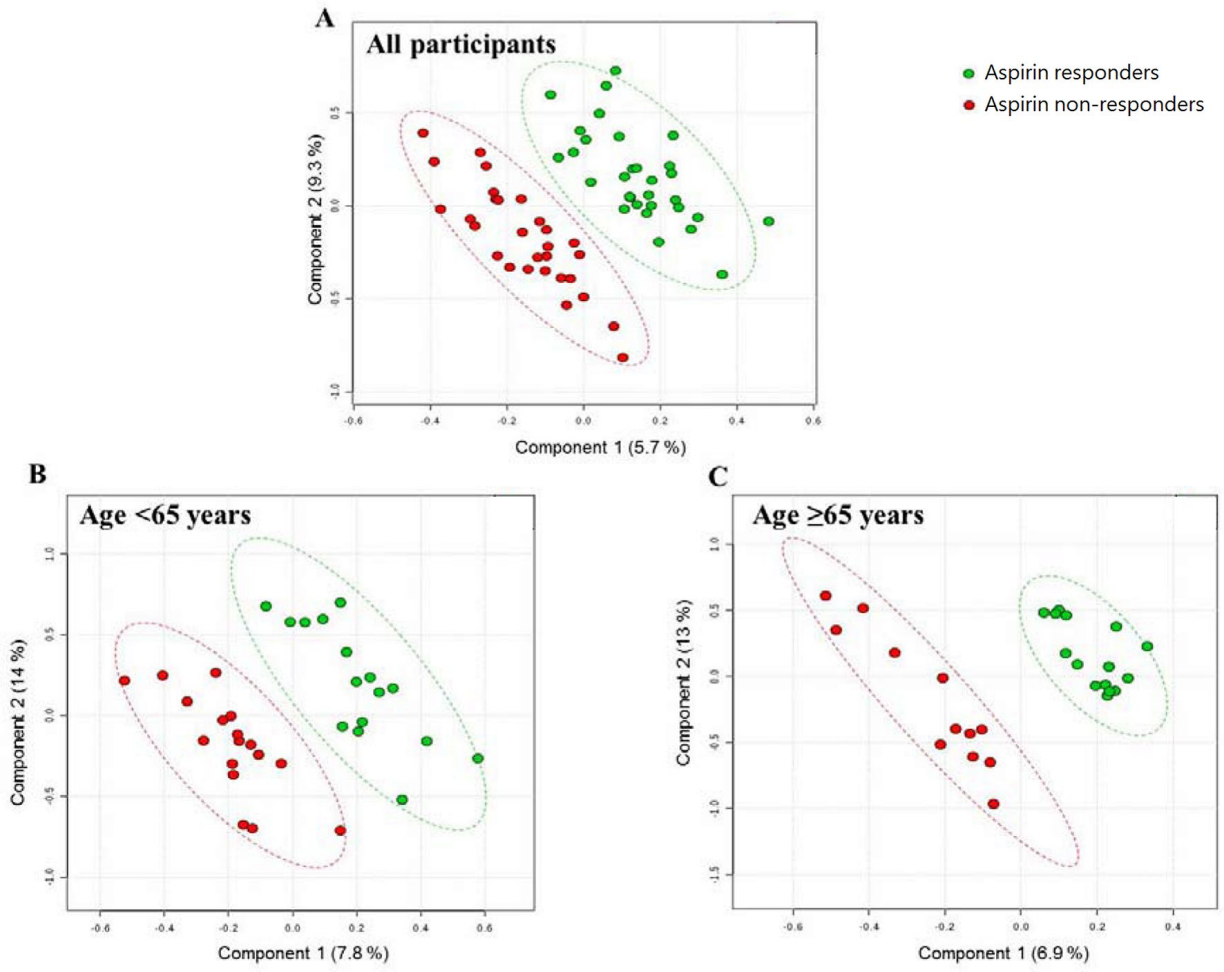
PLS-DA score plots from the analysis of p-JRES spectra using platelet samples. **(A)** Aspirin responder: n = 31; Aspirin non-responder: n = 29. **(B)** Age < 65 years. aspirin responder: n = 16; aspirin non-responder: n = 17. **(C)** Age ≥ 65 years. aspirin responder: n = 15; aspirin non-responder: n = 12. PLS-DA, partial least-squares discriminant analysis.

A representative NMR spectrum of hydrophilic platelet metabolites from one sample is shown in **Supplementary Figure 2**. A total of 11 metabolites were identified on the p-JRES spectum, including valine, lactate, alanine, glutamate, succinate, taurine, myo-inositol, glycine, ATP, ADP, and AMP. The PCA model from the analysis of platelet metabolome showed no clear separation between aspirin responders and non-responders before or after stratification by age. In the PLS-DA model, components were calculated to maximize the covariance between class assignment and the platelet metabolome. Each spot on the score plot denotes one sample. Aspirin responders and non-responders were slightly separated along both components (component 1 accounted for 5.7% of the total variance, and component 2 for 9.3%) in the PLS-DA scores plot (**Figure 1**). The predictive capability: Q^2^ of 10‐fold cross‐validation is 0.04; 1000 random permutation test is *p =* 0.99 (**Table 2**). Variable influence on projection of each metabolite was shown in **Supplementary Table 2**. After stratification by age (<65 vs ≥65), the metabolic profile was analyzed again, and the PLS-DA score plots revealed separated clusters between aspirin responders and non-responders. However, the cross‐validation was not ideal in those younger than 65 years (Q^2^ = −0.17; *p*_permutation_ = 0.99) and those 65 years or older (Q^2^ = −0.26; *p*_permutation_ = 0.990).

**Table 2:** PLS-DA models and permutation tests for distinguishing between aspirin responders and non-responders by age groups.

Population	Aspirin responder (n)	Aspirin non-responder (n)	PLS-DA		*p*_permutation_^b^
			Components^a^	Q^2^	
Overall	31	29	4	0.04	0.99
Age, < 65 years	16	17	4	−0.17	0.99
Age, ≥ 65 years	15	12	3	−0.26	0.99

PLS-DA, partial least squares-discriminant analysis.

Q^2^ = predictive capability of PLS-DA model.

^a^The number of components based on Q^2^ indicates the best classifier of PLS-DA using a 10-fold cross-validation method.

^b^1,000 random permutations performed.

### Association Between Candidate Metabolites and Aspirin Response

The levels of metabolites were similar between aspirin responders and non-responders in overall patients and in age-stratified patients (<65 years vs ≥65 years). There was no association between each candidate metabolite and aspirin response status after adjusting for confounders, such as sex and other comorbidities.

### Association Between Candidate Metabolite and Mean Clotting Time

When each metabolite was dichotomized into high and low levels by its median value, the clotting time was similar for all candidate metabolites. Because age is an important confounding factor, we further stratified this analysis by age groups (<65 years vs ≥65 years). We found that the clotting time was significantly different between high- and low-glycine groups (259.5 s vs 175.0 s, *p =* 0.007, **Table 3**). The glycine level was significantly positive correlated with the clotting time in this age group (**Supplementary Table 3**). However, this association became non-significant after correction for multiple tests. The median value of glycine among patients younger than 65 years was 0.002696. The sensitivity, specificity, positive predictive value, and negative predictive value of glycine level below 0.002696 to predict aspirin non-response was 0.77, 0.69, 0.72, and 0.73, respectively (**Supplementary Table 4**).

**Table 3:** The mean clotting time for high and low (median as the cutoff) level of metabolite by age group (< 65 and ≥ 65 years).

Metabolite level^#^	Clotting time (second), mean (SD)	
	Age, < 65 years	Age, ≥ 65 years
Valine		
High	224.0 (84.01)	211.9 (76.72)
Low	202.2 (84.33)	210.8 (95.81)
Lactate		
High	202.6 (84.27)	211.1 (80.15)
Low	230.1 (82.30)	211.6 (89.74)
Alanine		
High	223.6 (80.09)	210.9 (86.46)
Low	197.8 (89.62)	211.8 (86.38)
Glutamate		
High	218.4 (86.77)	203.4 (81.38)
Low	207.4 (82.17)	216.9 (89.17)
Succinate		
High	214.3 (89.43)	196.2 (85.53)
Low	212.8 (81.49)	233.5 (82.38)
Taurine		
High	213.9 (86.72)	225.8 (82.50)
Low	212.9 (82.94)	202.9 (87.37)
Myo-inositol		
High	227.6 (80.19)	210.3 (82.76)
Low	198.3 (86.70)	212.3 (89.17)
Glycine		
High	259.5 (68.33)	230.6 (76.57)
Low	175.0 (76.57)*	190.8 (91.17)
ATP		
High	205.1 (86.04)	211.6 (89.24)
Low	224.7 (81.89)	211.3 (84.48)
ADP		
High	238.7 (78.91)	210.5 (82.58)
Low	183.1 (81.25)	212.1 (89.30)
AMP		
High	231.5 (84.66)	225.9 (80.33)
Low	194.2 (80.56)	199.8 (89.10)

CT, clotting time; ATP, adenosine triphosphate; ADP, adenosine diphosphate; AMP, adenosine monophosphate.

^#^High and low groups of each metabolite were determined by the median level of corresponding metabolite.

*The significant p values are marked by *(p < 0.05).

## Discussion

This is the first study focusing on hydrophilic metabolites of platelet in aspirin non-responders by NMR-based metabolomics. Although our study showed that the metabolomic profiles were not different between aspirin responders and non-responders, a lower level of platelet glycine may be associated with shorter clotting time for patients younger than 65 years. By analyzing the results of PFA-100 from 100 healthy Han Chinese in Taiwan, we defined the normal range of closure time for collagen/epinephrine and collagen/ADP cartridges.

Investigation of platelet metabolomic profiles in aspirin non-responders is of great value, because the metabolic profile is closer to a disease phenotype than other upstream omics (Worley and Powers, 2013). Pharmacogenetic studies have demonstrated an association between gene SNPs and aspirin non-response, but these associations are controversial (Maguire et al., 2008). Certain miRNA is associated with aspirin non-response, but the exact change in metabolic pathway is unclear (Kok et al., 2016). By examining metabolic alteration, key metabolites can be identified, and proposed causes of aspirin non-response may be validated.

A comprehensive intracellular metabolic profile of platelets has been investigated (Paglia et al., 2014; Paglia et al., 2015). By Ultra performance liquid chromatography - tandem mass spectrometer, Paglia et al. identified 96 metabolites within platelets. Comparison between fresh and stored platelet concentrate using pathway analysis further revealed the most affected pathways during storage. These pathways included pentose phosphate pathway and nucleotide sugar metabolism. In another case control study, different platelet metabolism profiles between patients with and without Alzheimer’s disease were identified. A prediction model was constructed based on these differences (Oberacher et al., 2017). The results of these studies suggested that different platelet metabolic profile might offer new biomarkers in different diseases.

Thomas et al. conducted a systematic review of literature by collecting research on platelet proteomics and fluxomics (Thomas et al., 2014). They constructed a cell scale, mass, and charge balanced model of platelet metabolism, iAT-PLT-636. The metabolism of platelet in patients with aspirin resistance was analyzed with this model, and the researchers predicted increased glyceraldehyde 3-phosphate dehydrogenase and triose phosphate isomerase, and decreased 1,6-bisphosphate aldolase and reactive oxygen species *via* glutathione related pathways. As a result, flux through the pentose phosphate pathway and through glycolysis pathway increased, as did purine metabolism increased. Glycine takes part in the above three pathways. Glycine could affect platelet membrane potential and hence aggregation capacity by binding to glycine-gated chloride channels. Animal studies showed that dietary glycine significantly increased bleeding time of rats and decreased the amplitude of platelet aggregation (Schemmer et al., 2013). This evidence may suggest a potentially important role of glycine in the physiology of platelet activity. Our findings are consistent with this hypothesis by showing that lower glycine may be associated with higher platelet activity under aspirin treatment (**Supplementary Figure 3**). On the other hand, the correlation between glycine levels and aspirin non-response among patients older than 65 years is not significant. This implies a different mechanism of aspirin non-response in patients of different ages. In elderly patients, comorbidities, such as diabetes mellitus or metabolic syndromes, are commonly presented, and this may contribute to aspirin non-response (Wurtz et al., 2014; Du et al., 2016). Among our study population, the percentage of DM among patients older than 65 years is higher than those younger than 65 years. The presence of these comorbidities may attenuate the impact of glycine. Patients with sufficient platelet glycine might still be aspirin non-responsive given their background condition. This finding has important clinical implications since lower glycine might be a biomarker of aspirin non-response in younger patients, but not in the elderly.

There are some limitations in our study. Our study focused on hydrophilic metabolites of platelets, and glycine is the only metabolite that may be different between aspirin responders and non-responders in patients younger than 65 years. The hypothesized pathway change of aspirin non-response cannot be constructed. In addition, the small sample size potentially caused the statistical analysis to be underpowered. Since there is no research focused on this topic, our study might serve as a pilot study. Further research on hydrophobic metabolites or circulating metabolites, or with a different isotope, such as 13C, may provide more insights into aspirin resistance.

## Conclusion

In this pilot study, the hydrophilic metabolomic profile of platelets was not different between aspirin non-responders and responders. An association between lower glycine levels and higher platelet activity in patients younger than 65 years may suggest an important role of glycine in the pathophysiology of aspirin non-response.

## Perspectives Section

By metabolomic analysis, changes in the small molecules of platelets in aspirin non-responders may be detected, providing insights into the mechanism of aspirin non-response, and potentially discovering new biomarkers for aspirin non-responders.

- In patients younger than 65 years, a lower glycine level was associated with shorter clotting times
- Low glycine level may be a clinical biomarker for aspirin non-response in patients younger than 65 years.

## Ethics Statement

The study complies with the declaration of Helsinki and the study protocol was approved by the institutional research ethics committee (IRB number: 201106077RC). All patients gave written informed consent.

## Author Contributions

J-YChi, S-HL, J-MJ, and C-YL had full access to all of the data in the study and take responsibility for the integrity of the data and the accuracy of data analysis. Study concept and design: J-MJ, C-YL, Y-CC, S-HL, C-KW, H-MY, S-FY, J-YChu, S-CL, and F-TC. Acquisition of data: C-AH, B-BL, P-CC, C-HC, J-YChu, S-CL, and H-JL. Expert statistical consultation: S-HL, Y-CC, and C-YL. Analysis and interpretation of data: J-YChi, S-HL, Y-CC, C-YL, J-MJ, J-YChu, S-CL, and F-TC. Critical revision of the manuscript for important intellectual content: J-YChi, S-HL, C-YL, J-MJ, J-YChu, S-CL, and F-TC.

## Funding

The study was supported by the Ministry of Science and Technology (MST 102-2628-B-002-048-MY3 and MST 105-2628-B-002-026-MY3) in Taiwan.

## Conflict of Interest

The authors declare that the research was conducted in the absence of any commercial or financial relationships that could be construed as a potential conflict of interest.

## Abbreviations

AMI, acute myocardial infarction; TIA, transient ischemic attack; PAD, peripheral artery disease; PFA, platelet function assay; NMR, nuclear magnetic resonance; miRNA, micro ribonucleic acid; CAD, coronary artery disease; Col/EPI, collagen/epinephrine; Col/ADP, collagen/adenosine diphosphate; JRES, J-resolved spectroscopy; PCA, principal component analysis; PLA-DA, partial least squares discriminant analysis; ATP, adenosine triphosphate; ADP, adenosine diphosphate; AMP, adenosine monophosphate; DM, diabetes mellitus; SNP, single nucleotide polymorphism.
