# Supplementary material for: Metabolomic Analysis of Platelets of Patients With Aspirin Non-Response
Source: Front Pharmacol. 2019 Oct 10;10:1107. doi: 10.3389/fphar.2019.01107 (PMC6797853; doi:10.3389/fphar.2019.01107)
Supplement: Supplementary file 2 [file Table_2.docx]

Supplementary Table 2. The VIP values of the metabolites from the PLS-DA model.

| Metabolite | VIP value |
| --- | --- |
| ADP | 4.87 |
| Alanine | 10.34 |
| AMP | 2.25 |
| ATP | 9.32 |
| Glutamate | 4.07 |
| Glycine | 1.21 |
| Lactate | 21.53 |
| Myo-inositol | 2.97 |
| Succinate | 1.38 |
| Taurine | 19.02 |
| Valine | 0.52 |
