# Supplementary material for: Metabolomic Analysis of Platelets of Patients With Aspirin Non-Response
Source: Front Pharmacol. 2019 Oct 10;10:1107. doi: 10.3389/fphar.2019.01107 (PMC6797853; doi:10.3389/fphar.2019.01107)
Supplement: Supplementary file 3 [file Table_3.docx]

Supplementary Table 3. The correlations between the clotting time and glycine.

|  | Correlation Coefficient | *p* (two-tialed) |
| --- | --- | --- |
| All patients | 0.278 | 0.032 |
| ≧ 65 years old | 0.178 | 0.374 |
| < 65 years old | 0.390 | 0.025 |
