# Supplementary material for: Metabolomic Analysis of Platelets of Patients With Aspirin Non-Response
Source: Front Pharmacol. 2019 Oct 10;10:1107. doi: 10.3389/fphar.2019.01107 (PMC6797853; doi:10.3389/fphar.2019.01107)
Supplement: Supplementary file 4 [file Table_4.docx]

Supplementary Table 4. The sensitivity, specificity, positive predictive value, negative predictive value of low glycine level for aspirin non-response.

|  | all | >65 | <65 |
| --- | --- | --- | --- |
| Sensitivity | 0.66 | 0.5 | 0.77 |
| Specificity | 0.65 | 0.6 | 0.69 |
| Positive predictive value | 0.63 | 0.5 | 0.72 |
| Negative predictive value | 0.67 | 0.6 | 0.73 |
