# Supplementary figures and images for: Metabolomic Analysis of Platelets of Patients With Aspirin Non-Response
Source: Front Pharmacol. 2019 Oct 10;10:1107. doi: 10.3389/fphar.2019.01107 (PMC6797853; doi:10.3389/fphar.2019.01107)

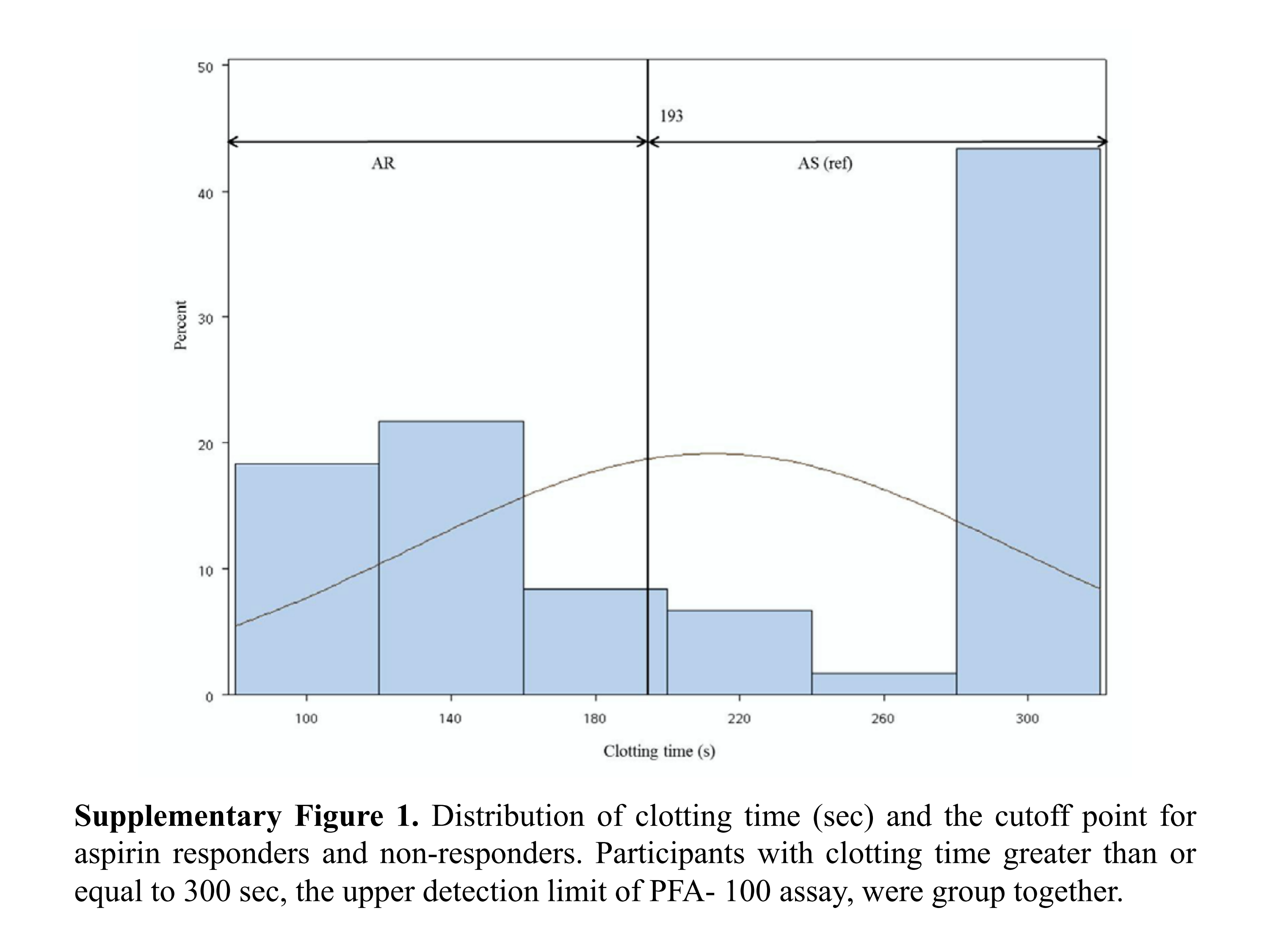

Supplement: Supplementary file 5 [file Image_1.tif]

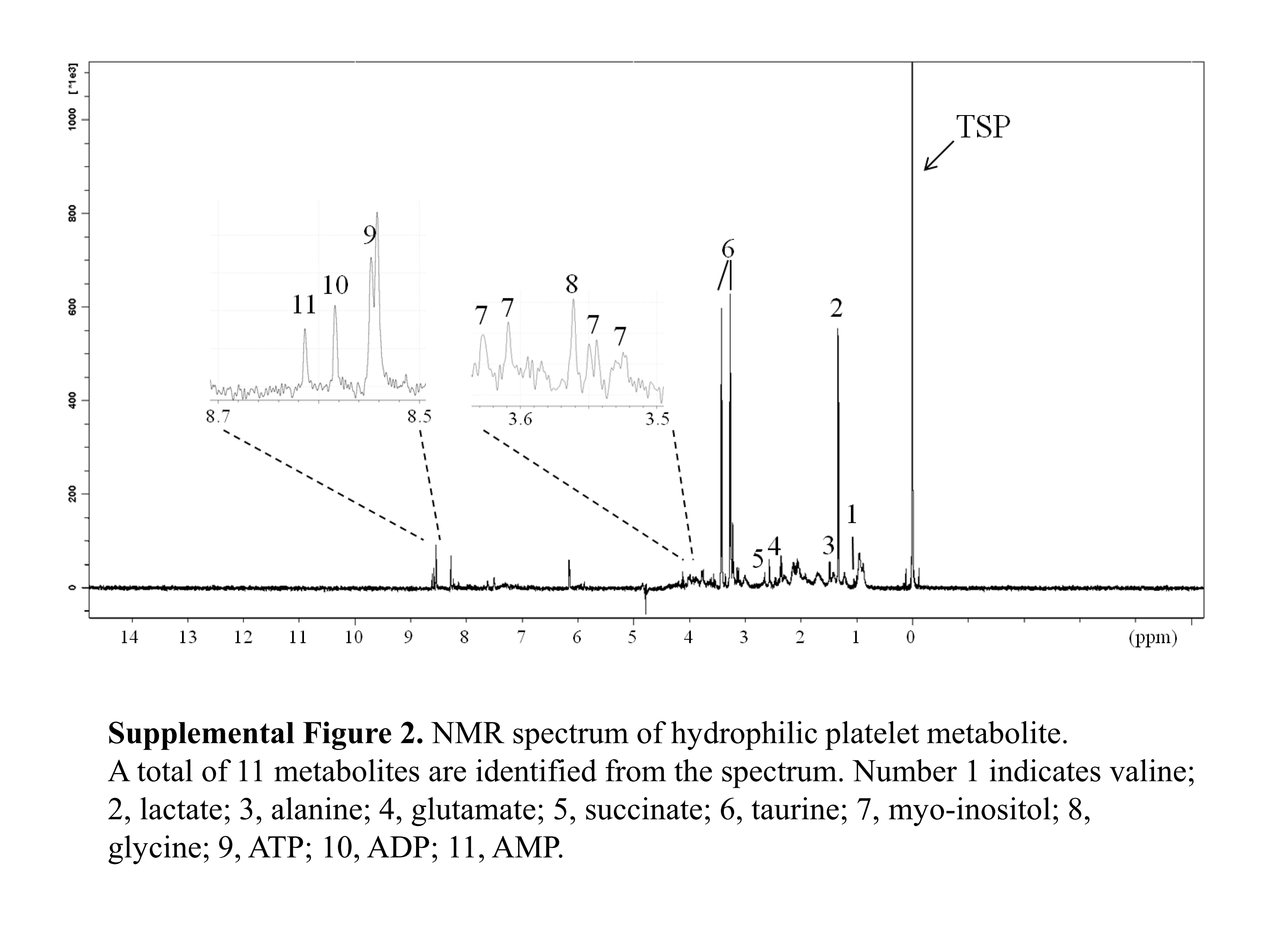

Supplement: Supplementary file 6 [file Image_2.tif]

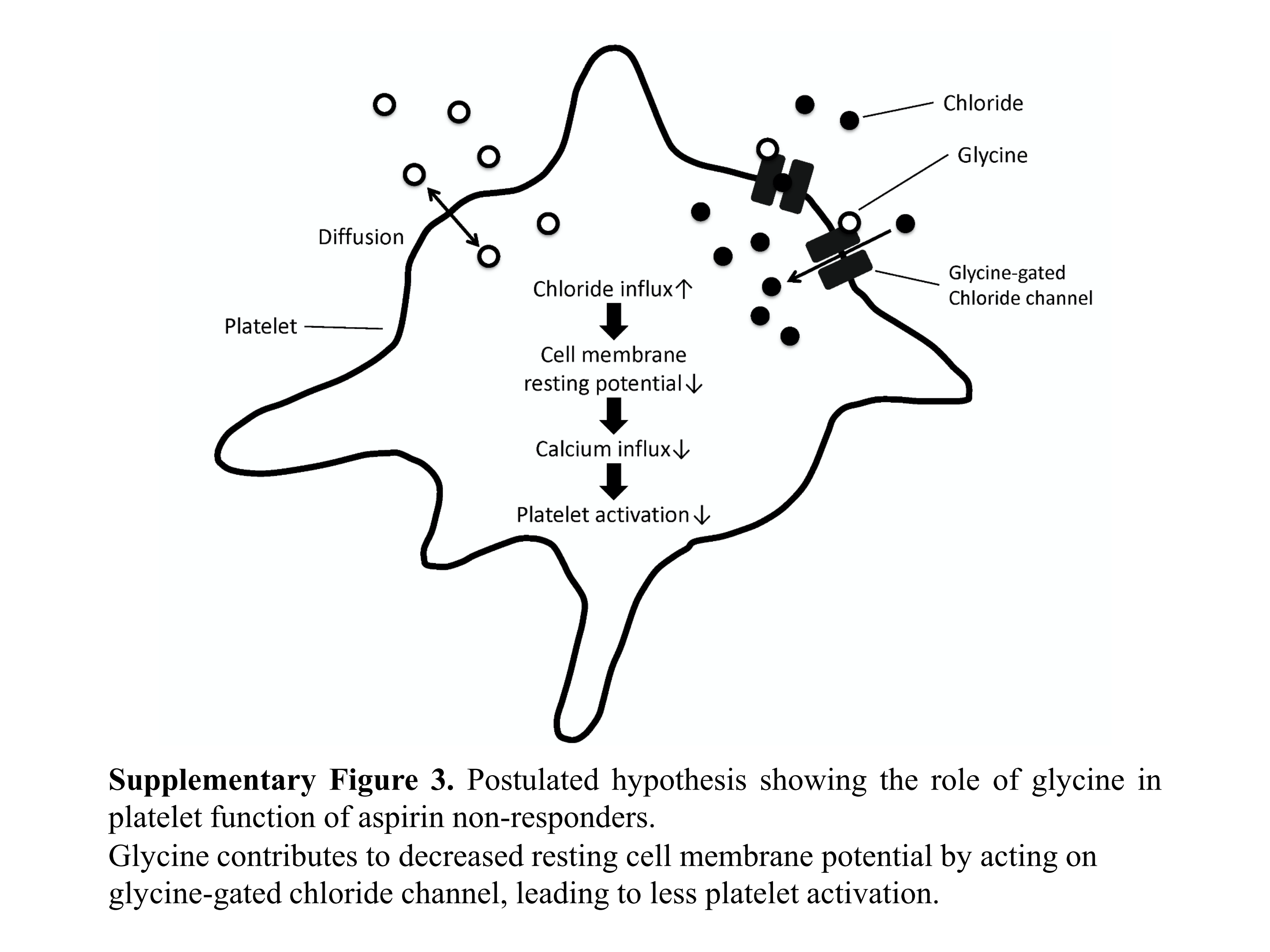

Supplement: Supplementary file 7 [file Image_3.tif]
